# Key factors influencing public health students and curricula in India: Recommendations from a mixed methods analysis

**DOI:** 10.1371/journal.pone.0279114

**Published:** 2023-02-09

**Authors:** Meike Schleiff, Haley Brahmbhatt, Preetika Banerjee, Megha Reddy, Emily Miller, Piyusha Majumdar, D. K. Mangal, Shiv Dutt Gupta, Sanjay Zodpey, Anita Shet

**Affiliations:** 1 Department of International Health, Johns Hopkins Bloomberg School of Public, Baltimore, MD, United States of America; 2 Indian Institute of Health Management Research (IIHMR), Jaipur, Rajasthan, India; 3 Public Health Foundation of India, Gurugram, Haryana, India; International Medical University, MALAYSIA

## Abstract

**Background:**

Building on a distinguished history of community medicine training, public health programs have been expanding in India in recent years. The COVID-19 pandemic has brought additional attention to the importance of public health programs and the need for a strong workforce. This paper aims to assess the current capacity for public health education and training in India and provide recommendations for improved approaches to meet current and future public health needs.

**Methods:**

We conducted a desk review of public health training programs via extensive internet searches, literature reviews, and expert faculty consultations. Among those programs, we purposively selected faculty members to participate in in-depth interviews. We developed summary statistics based on the desk review. For qualitative analysis, we utilized a combination of deductive and inductive coding to identify key themes and systematically reviewed the strengths and weaknesses of each theme.

**Results:**

The desk review captured 59 institutions offering public health training across India. The majority of training programs were graduate level degrees including Master of Public Health and Master of Science degrees. Key factors impacting these programs included collaborations, mentorship, curriculum standardization, tuition and funding, and student demand for public health education and careers. Collaborations and mentorship were highly valued but varied in quality across institutions. Curricula lacked standardization but also contained substantial flexibility and innovation as a result. Public sector programs were perceived to be affordable though fees and stipends varied across institutions. Further development of career opportunities in public health is needed.

**Conclusion:**

Public health education and training in India have a strong foothold. There are numerous opportunities for continued expansion and strengthening of this field, to support a robust multi-disciplinary public health workforce that will contribute towards achieving the sustainable development goals.

## Introduction

A strong health workforce represents one of the six building blocks of a health system as per the World Health Organization’s (WHO) framework. Skilled health professionals with sound technical and contextual knowledge are driving forces for national health systems [1]. Health professionals represent a crucial component of the supply side of health systems. Among the wide range of different professionals encompassed within the health workforce, cadres of workers focused specifically on public health play key roles in disease prevention, health education, surveillance, creation of policies, establishing and supporting engagement between communities and other stakeholders, evaluation of programs, and many other core functions [2,3].

The health workforce in India, outlined by Tiwari et al, consists of a range of clinical and non-clinical professionals, including doctors (allopathic as well as alternative and traditional medicine systems); nursing and midwifery professionals; public health professionals (medical, nonmedical); pharmacists; dentists; paramedical workers (allied health professionals); grass-root workers (frontline workers); and support staff. There exists within this ecosystem an interdependency that should also be reflected in training, career pathways and professional development opportunities [4,5]. Public health functions have been provided in India via both the Bachelor of Medicine, Bachelor of Surgery (MBBS) in preventive and social medicine and community health programs, which have been a well-established and important source of qualified workers for decades [6,7]. Community medicine as a discipline brings a public health approach to bear on clinical practice, and provides an opportunity to bridge between medical and public health programs [8]. In addition, PhD programs related to public health sciences have been available for some time.

India has reported one of the highest burdens of COVID-19 cases in the world, and its indirect effects and the national pandemic response has significantly challenged India’s health systems. Pandemic-related restrictions have led to disruptions in routine services for maternal and child health [4] and have halted the progress of communicable and non-communicable disease surveillance and treatment programs [9]. The pandemic has also highlighted the long standing and urgent need for universal health coverage (UHC) in India. Ensuring delivery of high-quality public health education, which can keep pace with changes in scientific, social, and economic spheres, is essential to meet India’s public health and universal health care (UHC) targets, particularly in light of the COVID-19 pandemic where public health has become a central factor in decision-making and priority interventions in India and around the world [10–13].

Supply and demand for public health training have experienced a noticeable uptick in recent years–including trainings all the way to master’s and doctoral degree programs [2,14]. For example, the number of institutions offering the Master of Public Health (MPH) programs in India increased from 2 in 1997 to 46 in 2017 [15]. However, previous work has identified that MPH curriculums were not standardized, nor overseen by an accrediting institution at the national or international level [14,16–18]. There remain high concentrations of professionals from medical backgrounds in public health training programs, which though important, leave many other potential entry points to public health such as social work, nutrition, nursing, and education among others underrepresented [1,10,17]. This relates to another necessary and valuable component of a strong public health workforce—a transdisciplinary, multisectoral approach—which is essential to addressing the broader social and environmental determinants of health; thus far, this potential has not been able to be fully leveraged [19,20]. Even with all of these important components of the public health workforce, a recent supply-side forecast for the number of public health professionals needed to support national public health interventions suggests that India is estimated at an additional 45,000 public health workers by the year 2026 [5].

The intent of this study is to better understand the current capacity for public health education and training in India. This is an important opportunity for India as the interest in public health has risen substantially in recent years as have a range of programs to provide these important skills. We describe opportunities and challenges across a set of key emergent themes related to the curriculum and the student audiences that are participating in available programs in order to identify priority recommendations and actions that can further catalyze the impact of India’s health workforce to address current and ongoing health challenges in India and globally.

## Materials and methods

**Fig 1:**
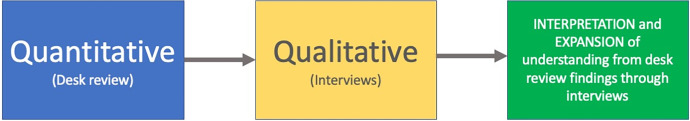
Study design, including use of each of the selected methods.

This paper utilized a sequential explanatory mixed-methods approach, including a desk review followed by a set of in-depth interviews (Fig 1). This study was reviewed and determined not to be human subject research by the IRBs at the Johns Hopkins School of Public Health (JHSPH) (FWA FWA00000287) and the Indian Institute of Health Management Research (IIHMR) (FWA FWA00018806).

### Desk review

#### Sampling and data collection

We conducted extensive internet searches to identify institutions which provide general or specialized public health training. The objective of the desk review was to gain an overall understanding the types, geographic distribution, target audience, etc. for public health training opportunities in India. We obtained a list of institutions using combinations of search terms that included “education” or “training” and “India" or each specific state in India (see S1 Appendix). Information on identified institutions were compiled to create a database of public health institutes (S1 File). We utilized the websites of the institution as the primary data source by looking through the academic department or program information and extracting data on key variables summarized in Table 1.

**Table 1:** Summary of variables captured in the desk review.

General information	Program characteristics	Student and faculty composition	Institutional collaborations
Name of institution	Type(s) of qualifications or training offered	Estimated number of students	Academic collaborations (India, international)
City(s) located	Core competencies	Number of faculty (fulltime, visit/adjunct)	
Tuition (including scholarships)	Main topic areas		
	Teaching modes (online, place-based)		
	Mentorship model		

During our initial round of searching, we captured institutions with a website presence that offered any public health related courses (diplomas, certificates, degrees) and included free-standing institutions of public health as well as programs of public health housed within other schools/colleges. From the initial round of searches, our team identified an initial set of 40 institutions. To ensure that we had captured all relevant institutions, we also reviewed literature including related capacity assessments and consulted expert faculty at institutions in India. This led us to include 19 additional institutions that offered MPH degrees that were not previously captured by our searches [5]. The final number of institutions captured through the desk review was 59 institutions offering public health courses and degrees. These included schools of public health, schools of medicine, and multi-disciplinary institutes. Community medicine trainings house within medical colleges were excluded from our study. Although these trainings constitute for a significant proportion of prevention-focused health activities in India today and throughout history, it has been noted in the past that these programs are more of an afterthought than a priority in these institutions, and that they have not evolved in pace with the discipline of public health.(7) The complete list of institutions can be found on the “Resources” page of Johns Hopkins International Vaccine Access Center webpage titled “Strengthening Institutions for Public Health Education: Table and Landscape Analysis”.

### Data analysis

We disaggregated the 59 institutions by the format of their public health training which included, stand-along certificates, Master of Public Health (MPH) (25); diploma (14); PhD (11); Master of Science (MS) (20); and skills-based trainings such as executive training certificate programs (3) or workshops (2). Institutions with multiple offerings were non-exclusively categorized for each respective offering. The desk review was presented both as descriptive and by training type. Programs captured in the desk review were categorized by type with each respective subcategory totaled. Key descriptors for programs were gathered when available and included core competencies covered in the program, estimated number of students, approximate number of faculty and tuition. Then, we constructed a representation for the geographical distribution of public health trainings in India by uploading each institution’s city into a custom Google map to depict geographical spread.

### In-depth interviews

#### Sampling and data collection

A series of 13 in-depth interviews were held with representatives from institutions identified by purposive sampling from the 59 institutions identified through the desk review using a semi-structured interview guide. The objective of the interviews was to discuss the findings of the desk review and understand both barriers and opportunities in practice for public health training in India. No respondents explicitly declined to participate, though we did not receive any response from all of those contacts. We aimed to capture a purposive sample of diverse perspectives from across India including public and private institutions, different kinds of training programs, and both long-standing and more recently established institutions and programs. We identified representatives via contact information available on the institutional websites, sites such as www.Shiksha.com that listed multiple programs, and via contacts made directly with faculty and leaders at these institutions that were previously known by members of the study team. Institutional representatives were invited by email to participate in an interview. In-depth interviews were conducted using online virtual conferencing platforms, which were recorded and later transcribed using a third-party transcription service (see Appendix 3 for the data collection tool) and were approximately one hour of duration. Study team members had prior exposure and practice with conducting qualitative interviews and familiarity with the Indian context, and a refresher training and practice session was conducted for this study prior to starting data collection. MR, PB, and HB conducted the interviews. All parties provided verbal consent at the onset of the interviews, as well as permission to be recorded and transcribed, with acknowledgment that participants’ responses would be anonymized; recordings and transcripts were also made available to interviewees upon request. The survey and interview guide were organized into a set of subject areas to capture key institutional qualities including general information, student and faculty characteristics, mentorship, funding, collaborations, institutional strengths, and challenges.

### Data analysis

Our analysis was based on a thematic analysis approach [21], drawing on both the study objectives to organize findings around the key research questions and also being sensitive to and inquisitive about new themes and connections emerging from the data. The transcribed interviews were uploaded into Dedoose (version 8.0.35, Los Angeles, CA, USA) qualitative data software and coded for analysis. Two members of our team created a codebook based on the semi-structured in-depth interview guide, which was shared with the entire team for initial review. We determined themes through a combination of inductive and deductive approaches. We established an initial codebook deductively that aligned with the research questions and associated interview guide themes. Four members of the study team then piloted the codebook with an initial set of transcripts, updating the codebook to reflect missing codes and/or discrepancies. All researchers had previous training and experience with qualitative methods. Once the codebook was finalized, the same four study team members divided the transcripts for analysis, with each transcript undergoing two separate analyses to ensure consistency. Then, during the coding process, as additional topics and considerations were identified, we discussed them as a study team and determined whether to create a new code and how to frame it. The final codebook consisted of 10 main codes and 32 sub-category codes. Regular meetings between the study members and note-taking accompanied this process to optimize organization and transparency. We aimed to explore both the strengths and weaknesses for each theme that arose as we recognized that each was complex with both pros and cons associated with it.

## Results

### I. Overview of public health education and training programs in India

Of the 59 institutions identified in our desk review, 32 (54%) were private institutions, 26 (44%) were public, and one was a public/private institution. Of among the 14 institutions for which we were able to identify this information, they had an average of 34 fulltime faculty members engaged in public health teaching.

**Fig 2:**
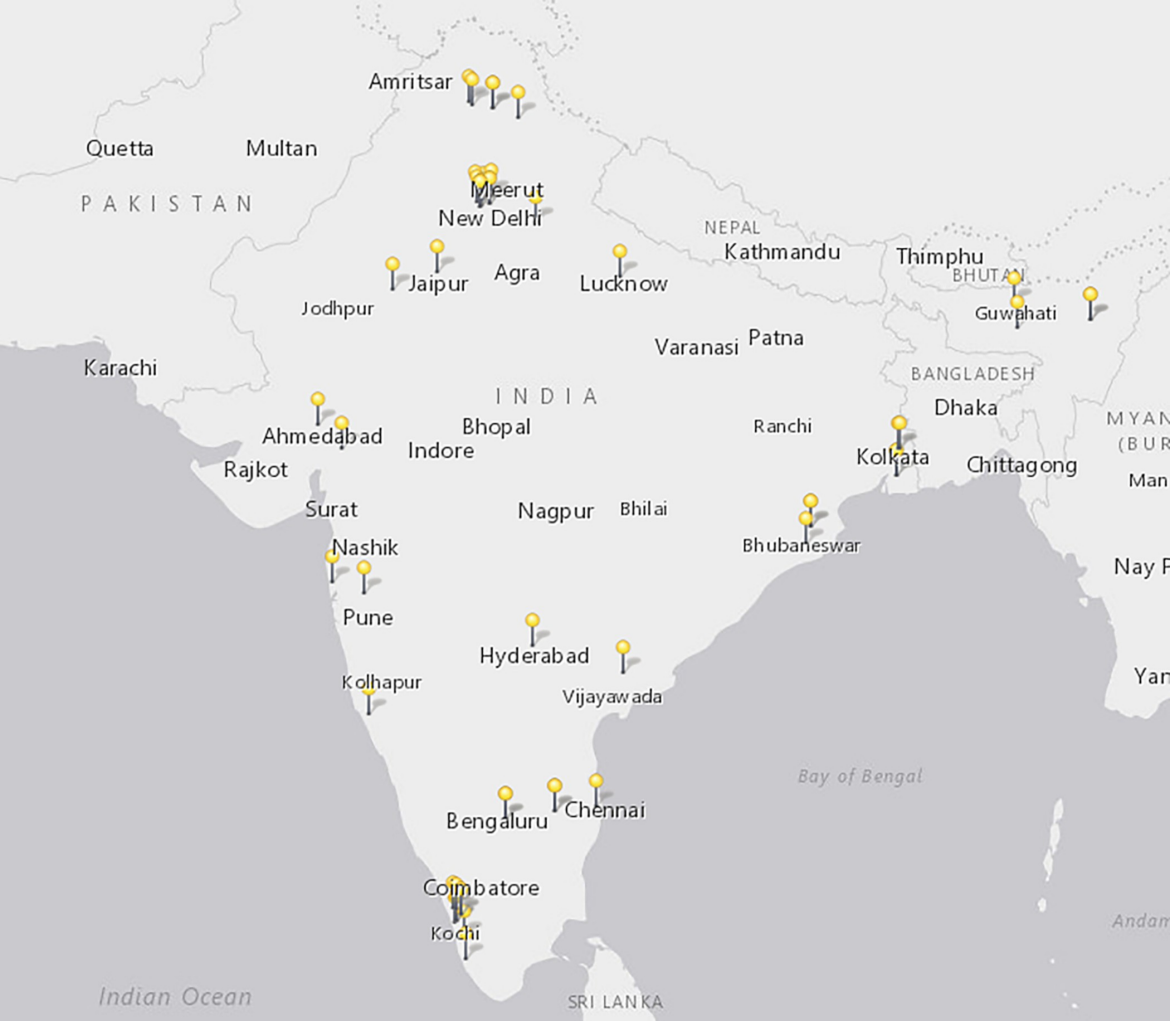
Map of institutions offering education and training programs across India.

Geographically, 45 cities across India were represented, with clusters in the North, Southwest and Northeast (Fig 2) including 11 programs in Kerala, eight in Karnataka, and seven each in Maharashtra and Delhi. We identified three institutions with multiple branches, and the two cities with the highest concentration of public health training were New Delhi (6) and Bangalore (5). Of the institutes identified, 19 states and 2 districts were represented. States with the highest concentrations of programs included Karnataka, Kerala, and Maharashtra (with 8, 8 and 7 programs, respectively). The district of New Delhi held the highest number of identified programs with 11 reported. Of the 11 easternmost states, only 4 states had programs identified (in West Bengal (3), Meghalaya (1), Assam (1) and Nagaland (1).

**Fig 3:**
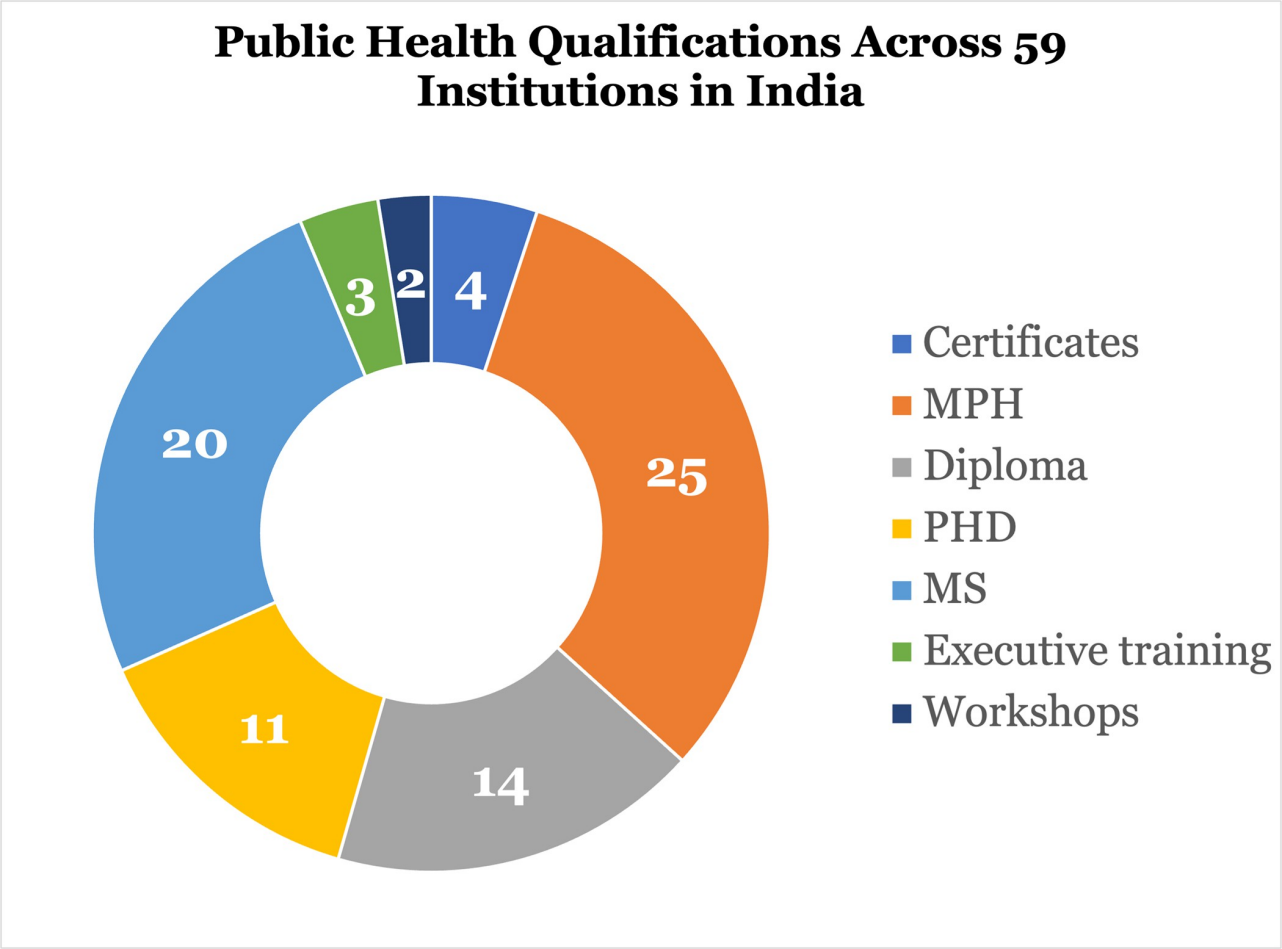
Public health qualifications offered across 59 institutions in India.

MPH programs constituted the most common kind of public health training opportunity in India with a total of 25, followed by Master of Science (MS) programs (20) and then diplomas (14) (Fig 3). PhD programs and a range of workshops and other certification opportunities were also available in smaller numbers.

**Fig 4:**
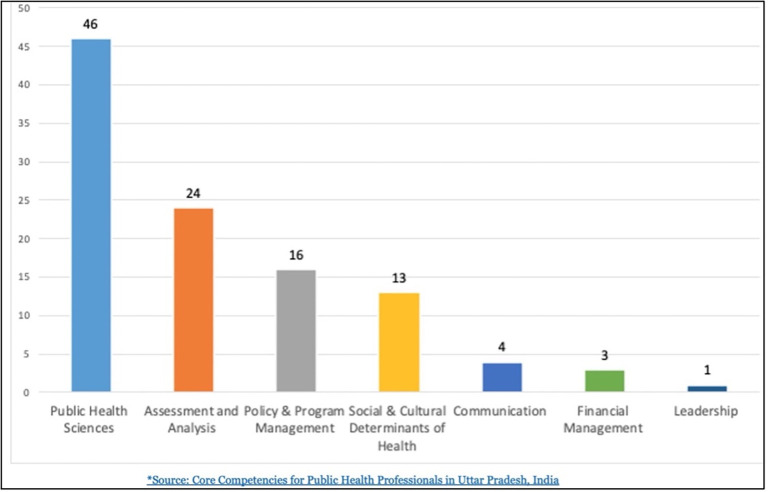
Frequency of core public health competencies addressed in public health training programs*.

We identified the broad knowledge, skills, abilities, and attitudes targeted by the different public health trainings offered, using a set of core competency domains for public health professionals developed in Uttar Pradesh, India.(2) We also adapted from a framework for the MPH programs that was previously developed for India [14]. Of the eight core competency domains considered necessary to deliver public health functions effectively by the Uttar Pradesh framework, public health sciences was by far the most common, represented in nearly all of the programs captured in this review. Leadership, communication, and financial management were the least represented in our desk review (Fig 4).

Beyond the competencies specifically covered in public health education and training programs, career and post-graduate outcomes of students trained in public health varied across institutions, several themes emerged from the in-depth interviews. In particular, the careers of public health students often relate very closely to their previous backgrounds resulting in path-dependency in public health training; once a student starts down one path of study, such as community medicine or non-medical field, it is challenging to change to an entirely different one, even as a result of specialized and in-depth public health training. One faculty program coordinator at a private university described the common clinical backgrounds that many successful public health careers are based on in India, which are often incentive by the government, as opposed to “… *the students who come in who are absolutely fresh and have just completed their bachelor’s or their master’s and have enrolled into a public health program”*. The former is more likely to have a set career pathway and identified job opportunities for which specific public health skills can add value whereas the latter often have a greater challenge identifying job opportunities and need an entire package of useful skills rather an a value-add to a well-defined field such as medicine. Thus, career trajectories for many students undergoing public health training are pre-determined according to their academic or professional background rather than being informed by the education they receive within a public health curriculum.

### II. Key factors influencing public health students and curricula in India

This section further describes key factors contributing to public health education and training curricula and students in India; these factors are summarized in Table 2 and the following sub-sections further describe and illustrate each of these themes in further detail. Factors related to institutional strengths and faculty capacity will be described in a separate manuscript.

**Table 2:** Key themes related to student body and curricula for public health education in India.

Theme	Strengths	Weaknesses
*Collaborations to support student learning*	• Students commonly engage with local communities and partners during training • These interactions are valuable for gaining skills and also informing longer-term career choices	• Availability varies across institutions and states
*Mentorship*	• Mentors support thesis development and often become more general career guides for students • Backgrounds of mentors are rich and variable; represent key leadership roles in the public health system	• Retention of mentors, particularly in rural areas • Areas for expansion • Formal structure or model can be lacking • Incentives and time commitment for mentors
*Curriculum structure and standardization*	• Room for flexibility, innovation, adaptation • ASPPH* competency framework has been utilized as reference in some programs; there is a Ministry-approved competency framework now	• Lack of standards/accreditation, • Competition for students and duplication of curricula between institutions • Challenge to ensure recognition or relevance of degree/diploma/certification
*Tuition and funding for students*	• Government funding supports a number of key programs • Tuition fees for public sector programs are affordable • Students can often earn stipends as part of their degree or by working	• Where tuition is more expensive and scholarships are limited, students generally rely on loans
*Student demand for public health education and career pathways*	• Students can get promotions after completing specialized trainings • MPH degrees are a positive feature for learners who want to go on and pursue a PhD	• Lack of clear career pathways • Lack of available job positions that focus on public health specialties particularly at the government level • Lack of motivation and incentives

*Association of Schools and Programs of Public Health.

### Collaborations to support student learning

*Strengths*: Institutions emphasized the need for and value of hands-on experience for students undertaking public health training. Specifically, many institutes used a practicum or field placement in collaboration with other organizations to provide field-based opportunities to students. One professor from a government institute explained that exposure to other institutions and their best practices is key to providing opportunities for learners to apply knowledge from their training programs into practice. Further, some institutes engaged in preexisting collaborations for students to gain first-hand experience working on government programs at local and national level to provide “*good insights of implementation of various national health programs with the help of the program manager*.” Some institutions have a systematic approach to assisting students with placements and these can include “*a placement team at the institution led by a faculty member supported by three faculty members and a program officer…*” as expressed by an interviewee working in a private institute. This approach would source placement opportunities and aid in matching students with appropriate placements.

These collaborations are entirely for the benefit of the student and are meant to further their skills-based education and are cultivated to supplement in-class instruction with hands-on learning opportunities. Institutions also emphasized the unique need for collaborations for students who may not have had direct field experience of public health programs and to “*attempt to train our students so that tomorrow in case they have a choice*, *they can serve at the primary health centers” as noted by a faculty working in a public-private institute*.*”*

*Weaknesses*: Collaborations for students vary for each institution and depending on whose perspective at a given institution is captured. Many students have opportunities to pursue placements in hospital settings or to engage in research, but experiences with public health practice and leadership are limited. One faculty member at a government institution explained these opportunities: *“We interact with the department of community medicine in the medical colleges*. *We interact with their faculty and they have students undergoing…the MD in community medicine*. *Our students go and interact with them and they learn*.*”*

Notably, international collaborations are often more highly valued or focused on than local collaborations with partners such as non-governmental organizations (NGOs) or public health offices. However, international opportunities for students are often unbalanced and create more opportunities for the international students to come have placement experiences in India than vice versa.

### Mentorship for students

*Strengths*: The two main functions of mentors were as course-coordinators and faculty mentors. The course coordinators were often identified as point persons who provided a range of problem-solving roles including dealing with logistics, administration, housing, and a range of other student needs. Faculty mentors were primarily identified as support for academic and professional guidance in order to help students develop their thesis as required by the MPH, PhD, or diploma curriculum. These faculty mentors were assigned based on topical knowledge and alignment with student interests. Students were expected to check in with their faculty mentors regularly as they develop their thesis, and one professor from a government university expressed: *“The mentor is the person whom they will contact almost every second day*, *third day*, *or at least weekly to update him or her on the progress*.*”*

Graduate alumni also served as mentors as expressed by an interviewee from a government research institute where they identified *“*…*graduates who are nearer to the place of posting or district of posting of a student*…*”*. An *“open style of mentorship” was offered to doctoral students*, *to facilitate any additional guidance as* one respondent from a government institute described, “*those on the DAC*, *the doctoral advisory committee*, *are also people whom we pick and choose as possible mentors*, *in case the PhD student requires or feels the need to*, *expand their mentorship beyond the supervisor*…*”*

The background of external mentors (those involved outside of the students’ university or organization) included government officers such as Indian Administrative Service (IAS) officials, public health policymakers, district and state health officers and faculty from other medical colleges and international universities. Having external mentors was seen as beneficial for student exposure and interest as one human resource management faculty from a private university said,

In fact, more recently, there has been a trend, in fact, of trying to get external mentors, you know, people who are completely external to the system, because, I think, at least for us, it’s actually an opportunity to learn different styles of mentorship… the latest [advances] in a particular field which I may not, for example be, necessarily an expert or be able to keep up with what is happening…it brings in new ways of thinking, it brings in, you know, new understandings, new perspectives.

*Weaknesses*: Many institutions identified challenges with existing mentorship structures. Turnover and lack of commitment from mentors was a common challenge described for sustaining mentors at institutions. The main reasons given for these challenges were a lack of time *“they are busy in their own jobs*,…*the time is not really committed”* Lack of incentives was also noted by a faculty in a government institute who explained that *“mentorship is sort of voluntary*, *we don’t pay anything to them”*. Only a few institutes recognized a formal structure for mentorship including written expectations for mentors. However, most institutions recognized the need to expand formal mechanisms for mentorship in their programs, and one interviewee from a government university explained that, “*mentorship as such is not a very*…, *good concept in India*,…*it is a very sweeping statement*,… *we generally don’t have a very formal mechanism*.*”* Interviewees also spoke about the need to focus on expanding faculty mentorship beyond a student’s thesis project and onto other academic areas, and on improving delivery of mentorship during COVID-19, particularly through building on online mentorship platforms as one private university faculty said, *“with technology that has improved now*,…*we will improve upon that [for] providing mentorship over distance*,…*and through online media*.*”*

### Curriculum structure and standardization

*Strengths*: Enhancing curriculum structure and innovation through exposing students to experiences and opportunities abroad was mainly described for dual PhD programs where students spend their time abroad or where institutions expand their course modules with international universities. This curriculum exchange was expressed by a government institute faculty who said, “*we were exploring*…, *doing a dual PhD program*, *or*… *having an exchange with*, *say*, *a couple of modules in their MPH that we don’t teach*, *and in our MPH that they don’t teach*.*”*

*Weaknesses*: One challenge related to public health training in India was the lack of standardization in the curriculum and oversight across institutions offering public health education (PHE). A lack of having a structured curriculum was identified to affect the quality and value of public health education that students obtain in certain institutions as expressed by a government institute professor as, “…*in India unfortunately*, *there are small institutions who have started their own MPH because there is no regulation*, *and nobody really knows what they are churning out*.*”*

In the absence of a centralized framework or oversight of public health education, curriculums were sometimes identified as to be lacking necessary high-quality components, for example from disciplines like social sciences. As one interviewee from a private university said, *“the academic rigor that needs to come… into a public health program is usually a little lower in India from every single program*… *and it stems from a historical neglect of the social sciences by medicine*.*”* Other faculty also pointed to an overly focused medical lens to public health training as well, indicating this was not particular to their own institution but across the field of public health. Standardization was deemed necessary to address the broad nature of public health and overcome the extensive medicalized view as pointed out by a private institute faculty who said, “*I’m a doctor myself*, *but we have a very medicalized point of view towards addressing public health… There is a standardization of what should be covered*…*to get that multidisciplinary pool who can appropriately deliver the content is also going to be immensely important here in this setting*.*”*

Others also expressed concern that the scope of training was not necessarily representative of the skills or knowledge necessary to address real-world scenarios and there was a need to identify a standard framework to address this translation of public health skills as one from a government university said, “*The focus has been about*,…*developing technologies to make health accessible*. *But to really understand*,…*what this means*…*to really see how the potential for how all this works*, *a public health framework is really needed*.*”*

### Tuition and funding for students

*Strengths*: Almost all respondents made note of how funding impacts students and public health training as on interviewee from a government-affiliated institution noted, “[for public institutes] *the grant is from the government*. *So*, *there is no problem*, *as far as the funding is concerned to our institute*.”. In particular for many institutes funding was not a notable barrier specifically for public universities and government affiliated institutions as noted by one faculty, “*the MPH course*, *we used to offer till this year it’s free of cost to all the students*.*… All the students who are sponsored by the state health departments*”. Tuition fees are often nominal as opposed to private institutes where tuition rates are higher. There are also other forms of support from public institutions. As a respondent from a government institution said, “*the fees here [are] very*, *very nominal…there are no scholarships*, *rather to your surprise you will find that we actually pay our students a stipend because they are residents*, *so they get junior residency*, *which is a substantial amount…one-month junior residency can take care of their whole year’s fees*.”

*Weaknesses*: In general, funding was not recognized to be a barrier to student entry, even in the case of private institutions where tuition is considered to be higher, as the draw for highly reputable institutions and post-education opportunities outweigh higher tuition rates in student program selection. One respondent from a private institution explained,

Our fee structure for courses in health and in hospitals is higher… compared to other universities or institutions or schools offering similar programs in the country. But still, we are able to attract people. Usually, our stake is almost 100% full every time and the reason for this is our reputation.’

Scholarships at such institutions were limited, and students seeking financial support are expected to rely on private loans from banks and, in some cases, foundations.

### Student demand for public health education and career pathways

*Strengths*: For students who already have work experience in the health system in India, they often have a given position in mind prior to entering the program including being nominated by the government to participate in particular programs. One respondent described an example of this saying, *“They [a particular group of students] are basically the health educators… So*, *after*, *you know*, *joining this course*, *they get a promotion also*. *So*, *more than 50% of our students*, *they are the in-service candidates*.*”*

Other common targets for graduates aside from the health system were other government agencies, non-profit organizations, think tanks, industry-oriented companies, and further education. One private institute noted that demand changes depending on where in your career you are:

Almost immediately, after MPH, most individuals take a research officer course, research associate, that could be one important group of people. And then, there are a few who directly join program officer type implementation posts in government programs at the district level, [or] at the state level.

Some students moved forward to pursue their PhDs not only in public health but in other fields of science as one interviewee at a private institute said, *“there are others who’ve gone on to do PhDs in the basic sciences*, *and the engineering sciences and in public health and then there are others who have become very leading figures in government*.” Students also used their public health degree to branch out to other areas such as healthcare technology in large companies. The same respondent also described how this works: “*…lots of people who have gone to*, *you know*, *industries like Siemens*, *and GE*, *and Philips and Centricity*… *that work on different technologies in the health sector…*” Finally, another private institute respondent recognized that the MPH degree was a significant value for students who would want to pursue public health careers internationally: “…*MPH as we know*, *one of the programs that they find a very useful as a steppingstone to further employment opportunities abroad*.*”*

*Weaknesses*: In order to pursue public health, a government institute faculty member noted that students need to be assured that they will have “*a regular job”* and *“[a] basic education qualification through which I [student] can seek an employment…”* once they graduate. Experts interviewed also identified the current lack of job positions and career growth opportunities in the Indian public health sector which needs to be improved to trigger greater demand and increase student enrollment in public health training programs.

One interviewee from a private institution, while speaking on the value of public health degrees in India said, “*the promotion or the career advancement doesn’t happen automatically*… *even if you get [an] additional degree*, *in many states it really doesn’t matter”* indicating the apprehension for students to enroll in public health training without proper career incentives and clear pathways to additional opportunities once earning their qualifications.

While speaking about the lack of public health job postings in India, interviewees also expressed the clinical dominance of the field in India, such as this faculty program coordinator from a private institute who stated that in order to secure placements and be eligible for more career avenues and to increase demand for public health students. Particularly, one interviewee from a private institute spoke about the need to expand current postings in the government sector onto public health specialties in order to create demand: *“[governments]have not*…*expanded into the specialty in*… *public health… And that’s why the demand for the post is also not there*.*”*

Going beyond creating demand among students to enroll in public health programs, a need was identified to target the existing workforce that performs public health functions and offer a basic level of education as well as continuing education and training opportunities. As one interviewee from a private institute explained, “*At the level of the other rungs of the*… *public health cadre*… *ASHA*, *ANM’s*, *nurses*… *there has to be a planned strategy for*…*their basic education*, *but also for their continuing education*.*”* This need to strengthen the existing workforce also relates to another challenge of making public health training programs equitably accessible to students who can benefit from them and contribute to the Indian health system. Another private institute respondent described this challenge as, “*the idea was to open up opportunities in a way that is equitable*, *and there have been challenges to doing this*. *But they have really tried to figure out how to do this across the history of these institutions*.*”*

There was an identified need to provide more provisions for students to go abroad “*as a part of their elective program or the clerkship program” (private institute faculty member)*. The rationale for these opportunities is to expand on and enhance training offerings that were currently identified to be unstructured or limited, and to provide students with more opportunities for careers and diverse field work experiences so that they are prepared to work directly with communities. Other than the lack of available career growth opportunities, one interviewee identified a lack of amenities in their institution as a deterrent to student enrollment, “*being in the rural area is a weakness also; people don’t want to come [for training] because we have minimum physical amenities*, *facilities” (private institute faculty member)*.

## Discussion

Through this landscape analysis, we explored the already substantial capacity and many strengths for a range of public health education and training opportunities across India. Building on previous efforts to assess the number of master’s programs and define some core competencies for public health in India, we aimed to explore further the diversity of programs available, how these programs are working, what the curriculum covers, and what the strengths and challenges are for the students currently enrolled in them.

Several key findings include that Master’s level training is the most common qualification in public health that is available, but the expansion of diverse trainings has provided opportunities to experiment with different kind of programs and reach a broader range of students. We found that field-based placements are valued by students for job preparedness by offering real-world experience and skills-focused training, yet the quality and availability of partnerships varied across institutions. Tuition was generally not seen as a barrier, while that lack of clarity of career pathways, particularly for students without a prior medical background was a substantial barrier.

Our findings are bound by certain limitations. Our search only captured institutions that had a website presence or that had been captured in previous reviews; therefore, it is possible that we missed other institutions that are offering public health education and training but were not possible for us to identify for this desk review. We aimed to maintain regular and in-depth communications among the study team, particularly during the data coding period in order to achieve shared understanding of the data and the coding process, and to conduct member-checking as needed with respondents in order to ensure that we were interpreting the findings of the study appropriately. However, these methods are not without their limitations and we recognize that we may not have been able to entirely capture all nuances of these programs within this study. We explicitly did not include community medicine programs so that we could focus on the recently growing offerings of programs focused explicitly on public health as these are evolving and understanding these can provide new insights and identify opportunities for further growth and innovation. We do, however, recognize the invaluable contributions and long history of community medicine related to public health in India. We also faced challenges in contacting individuals across institutions largely due to COVID-19; therefore, we were not able to capture additional quantitative data from all institutions that were identified in the desk review as was originally our plan. We also did not capture direct perspectives of students or supervisor and mentors in the workforce; these components are part of a second phase of this landscape analysis and will be forthcoming in subsequent manuscripts. Notwithstanding these limitations, our findings provide several actionable insights and reinforce known principles about ways that public health training is currently taking place in India and how it can further contribute to the health workforce in India and beyond.

Public health curricula must be designed with a focus on meeting current demand, capturing broad determinants of health and providing sufficient guidance for career pathways and active public health practice post their completion [10]. We found that a range of training opportunities do exist, but that the direct linkage with career opportunities is not consistent or sufficient yet. Student intake data for public health programs were not consistently available online during our data collection process, so we could not include this in our analysis; however, previous studies have estimated annual capacity for MPH programs in India at 850 students per year, whilst also acknowledging historical enrollment rates around 75% of capacity [22,23]. Key to overcoming this challenge is that public health as a profession needs to be further recognized, including within the public sector workforce in India. One key aspect to advancing this goal is to have clear incentives and career pathways for students with public health qualifications are lacking. Importantly, our findings reinforced that a public health workforce with multidisciplinary skills would strengthen the health workforce and reduce the focus on medical skills alone [19,20].

Another observed limitation of public health education in India from our study is a heavy reliance on training programs housed in and built around medically focused institutions and curricula. Historically, the framework for preventative health interventions relied on principles of hygiene and a biological lens to pathology; this led to a paradigm in which public health education trainings are housed largely within medical colleges and taught by faculty from a medical background [6]. In general, students who pursue public health training and jobs in India still have a prior clinical background. While training clinicians in public health is very important, further diversification beyond this audience is necessary in order to expand the workforce and bring additional skills and perspectives to bear on public health programs [5,24].

Expansion of a workforce with strong public health training is also needed in order to continue to propel India towards its UHC goals [25] and to fulfill the 2017 health policy objectives which include major public health-oriented goals including improvements in data systems, increased health service coverage rates, and financing and infrastructure goals [26]. Notably, human resources priorities for the 2017 health policy do not explicitly emphasize strengthening the health workforce but focus on primary care clinicians and community health volunteers [26]. In addition, India ushered in a new National Education Policy (NEP) in 2020 that provides a framework for secondary education with multiple levels including one-year certifications, two-year diplomas, four-year bachelor’s, and the establishment of a Higher Education Council of India that will regulate higher education [27]. Both of these recent policies provide further structure and objectives that can guide public health training curricula moving forward.

## Conclusions

We set out to understand the current capacity, key considerations and needs for students and public health curricula in India. Public health education and training is well established in India, and numerous opportunities exist for continued growth and contribution to India’s development and resilience. Our findings yielded important and complex themes, that can inform decision-making and resource allocation going forward. First, a focus on multi-skilled workforce and multi-disciplinary training approaches should be the cornerstone of public health training programs. Second, sustained advocacy for public health careers and the value of public health skills is critical. Third, credentials offered at the end of courses must be recognized by local employers, including the public sector, and should be strategic for learners who want to pursue additional public health training. Fourth, mentorship is critical for ensuring that trained workforce remain contributing in their fields and should be strongly encouraged and incentivized in order to sustain motivation in public health work. Finally, faculty and learner feedback should be effectively integrated into curricula and student support programs to ensure that training programs are relevant and effective. Further alignment of educational opportunities with the job market and recognition of the value of public health skills are needed in tandem with continued curriculum enhancement to ensure an even stronger public health workforce for the future in India and the broader advancement of global health.

## Supporting information

S1 Appendix (DOCX)

S1 File Blank desk review sheet used to create the database of public health institutes. (XLSX)
