# Supplementary material for: Key factors influencing public health students and curricula in India: Recommendations from a mixed methods analysis
Source: PLoS One. 2023 Feb 9;18(2):e0279114. doi: 10.1371/journal.pone.0279114 (PMC9910711; doi:10.1371/journal.pone.0279114)
Supplement: S1 Appendix — (DOCX) [file pone.0279114.s001.docx]

**Appendix 1: Search Terms**

Google

“India” AND “public health” OR “master’s in public health” OR “community health” OR “health diploma” OR “population health” OR “public health school” OR “public health institution” OR “global health institute” OR “public health training” OR “community health training” OR “public health curriculum” OR “public health student”

**Appendix 2: Interview guide for in-depth Interviews**

**Interviewee Socio-demographic**

Participant ID:

Designated Institution:

Target Audience category:

Gender:

Age:

Participant Education:

**Introduction**

*Thank you for taking the time to speak with me today.  You/a representative from your organization responded to a survey that detailed a lot of information about your program. It was very useful.  I have some additional questions that I’d like to discuss with you, on how your institute or program was started, how it has evolved and who your current students are, and what you think the strengths and weaknesses are.*

**Interview Guide [Organized by Subject Area]**

| **Subject Area** | **Key Questions with Probe** |
| --- | --- |
| General | 1. To start, please tell me when you joined your program/institute, and what your current role is. |
| Students | 1. What do you think your students are most interested in gaining from your institute or program?      1. What kind of roles do students who complete your training programs go on to pursue? 2. What opportunities do you provide your students for effectively translating skills gained in school to real world application?   Probe:   - What kinds of periodic career workshops and fairs do you conduct? - How are students allowed to/encouraged to participate in field placements and/or conferences? |
| Mentorship | 1. What role does mentorship plan in courses and degrees at your institution? 2. What kinds of opportunities do you see to expand on the mentorship provided to students?   Probes:   - Does your institute allow for external mentorship on student projects? If no, do you plan to allow such mentorship in the future?      1. What impact do you think doing more mentorship could have on the capabilities, motivation, of the public health workforce in India? |
| Faculty | 1. How has your faculty composition changed over time? 2. Does your institution encourage/sponsor faculty to participate in exchange programs/sabbaticals in other local/national/international institutions to build on mentorship skills and forge new collaborations? 3. What do most of the faculty spend their time on?   Probes:   - Is your faculty expected to spend more time on research or on their teaching duties? - Do your faculty contracts allow for faculty to simultaneously work on projects outside your institution while they hold a teaching position? - Does your institute fund/conduct research that is headed by the faculty? What sort of research is this? Is your faculty expected to bring in external funding for their projects? - How much time are your faculty allowed off from their teaching duties for research? |
| Funding | 1. Regarding funding, how much does tuition contribute to the resources needed to sustain your institution?      1. What has worked best in terms of fee structures to attract high quality students and make programs viable in terms of efficient management and financial sustainability?      1. Is there anything else about tuition (what was worked well, challenges, what is needed in the future) that you would like to share? 2. What kind of scholarships or financial support, if any, does your institution offer to students? |
| Collaborations | 1. How much collaboration does your institute do with local and national partners?  International?      1. How have your collaborations changed over time?      1. What are the collaborations that you are most proud of? 2. Does your institute engage with local communities for any aspect of its programs/ initiatives?   Probe:   - What are some of these programs/ initiatives? - What does the relation between the institute and the community look like?  1. What are you seeking through a collaboration? 2. How often are you approached and how often do you approach other institutions? |
| Institution Strengths | 1. What are some of your institution’s/program’s greatest strengths?      1. What topics or competencies do you most excel at for public health workforce training?   Probe: What contributes to your institution’s excellence in this competency?   - Prompts—MCH, epi, bio, health systems etc.  1. What makes your institution stand out amongst its peers? |
| Challenges to and Future of Institution/ Program | 1. Where do you think your institute/program should aim to build further expertise?      1. What are some threats to the future and growth of your institution/program?  - Prompts—COVID-19, social movements, threats to vaccination      1. Where do you see the most change/growth happening over the next five years?  - Prompts—epi, bio, health systems etc. |
